# Lane Position Detection Based on Long Short-Term Memory (LSTM)

**DOI:** 10.3390/s20113115

**Published:** 2020-05-31

**Authors:** Wei Yang, Xiang Zhang, Qian Lei, Dengye Shen, Ping Xiao, Yu Huang

**Affiliations:** 1 College of Automotive Engineering, Chongqing University, Chongqing 400044, China; 201834131027@cqu.edu.cn (Q.L.); 20142572@cqu.edu.cn (D.S.); 20190702089t@cqu.edu.cn (P.X.); 20193202037t@cqu.edu.cn (Y.H.); 2 School of Information, Zhejiang University of Finance Economics, Hangzhou 310018, China; zxiang@zufe.edu.cn

**Keywords:** lane line detection, lane line prediction, long short-term memory, recurrent neural network

## Abstract

Accurate detection of lane lines is of great significance for improving vehicle driving safety. In our previous research, by improving the horizontal and vertical density of the detection grid in the YOLO v3 (You Only Look Once, the 3th version) model, the obtained lane line (LL) algorithm, YOLO v3 (S × 2S), has high accuracy. However, like the traditional LL detection algorithms, they do not use spatial information and have low detection accuracy under occlusion, deformation, worn, poor lighting, and other non-ideal environmental conditions. After studying the spatial information between LLs and learning the distribution law of LLs, an LL prediction model based on long short-term memory (LSTM) and recursive neural network (RcNN) was established; the method can predict the future LL position by using historical LL position information. Moreover, by combining the LL information predicted with YOLO v3 (S × 2S) detection results using Dempster Shafer (D-S) evidence theory, the LL detection accuracy can be improved effectively, and the uncertainty of this system be reduced correspondingly. The results show that the accuracy of LL detection can be significantly improved in rainy, snowy weather, and obstacle scenes.

## 1. Introduction

In order to avoid road accidents and ensure the safety of driving, in recent years, the research and development of the advanced driving assistance system (ADAS), which provides help for drivers, is booming all over the world [1,2,3,4]. Lane departure warning and lane keeping, lane change and forward collision warning, adaptive cruise control, and blind spot detection systems all belong to the category of ADAS. Among the many assisted driving technologies, LL detection is the core part of these systems [5,6,7]. Therefore, it is of great theoretical value and practical significance to study LL detection.

At present, LL detection is still a difficult research topic. In the real world, the structure is complex. Different countries have different traffic laws and regulations, and LL signs are different. Even in the same country, due to the differences in landform, the types and ways LL signs are used between cities are different. The differences of these types of markings are should be overcome by the algorithm itself. In addition, the LL is easily corroded and blurred with time, making it difficult for some algorithms based on computer vision for detection [8,9,10,11,12]. Furthermore, weather is also an important factor affecting the accuracy of the LL detection algorithm. When snow and rainstorms occur, LL is easily blocked, and visual detection of LL signs cannot be implemented at all [13]. Some other rule-based methods are needed to solve LL in similar scenes.

In the current LL detection algorithms, there is little research on the spatial distribution law of LL. However, LL has a clear distribution law in the structure, and the same type of LL sequence exists in the front of LL with high probability. Moreover, the distribution law is not affected by weather, the visual field, or obstruction. This study intended to establish an LL prediction model based on Long Short-Term Memory (LSTM) [14] and recurrent neural network by analyzing the distribution relationship of LL on the road surface. Only part of the known LL coordinate information is needed to predict the location of other LLs in complex scenarios.

The grid size of the bounding box is S × S in the YOLO v3 [15]; however, this density of grid cannot be applied for the detection of such a small size and large aspect ratio targets, like LLs. In our previous research [16], by improving the horizontal and vertical density of the bounding box in YOLO v3, the obtained LL detection algorithm YOLO v3 (S × 2S) improved the accuracy of LL detection. However, when the LL is occluded, the accuracy of the algorithm is poor. This research is a continuation of the problem of LL detection. On the basis of YOLO v3 (S × 2S), we further studied LL detection in complex scenes. The main contributions are as follows:

1. The use of a YOLO v3 (S × 2S)-based LL recognition model to detect LL position information and construct an LL position sequence;
2. Based on the historical position information of the detected LLs, a prediction model (ALSTM-RcNN) combined with angle information and a recurrent neural network, respectively, is presented.
3. To ensure the accuracy of the final LL detection results, the D-S evidence theory is used to integrate the output of the detection model and the prediction model to obtain the optimal position information.

## 2. Related Work

To reduce the influence of environmental factors on the detection algorithm, researchers use schemes that combine cameras, radars, high-precision maps, and so on to improve the system’s lane detection performance under complex operating conditions. Shin et al. [17] proposed an LL detection algorithm using a combination of cameras and Light Detection and Ranging (LiDAR), which used images and radar information to improve the LL recognition capabilities in harsh environments. Rose et al. [18] integrated cameras, Global Positioning System (GPS), and LiDAR to estimate the distance between the vehicle and lateral LL. De Paula et al. [19] proposed a lane detection algorithm using LiDAR, where a local grid reflection map around the vehicle was constructed using radar, and the LL was extracted from map information. Jung et al. [20] proposed a real-time model for lane detection using LiDAR data, which classified drivable areas, identified road signs, performed lane detection, and updated the corresponding parameters in real time. In combination with the GPS system, the detected road parameters were integrated to build a lane-level digital map. Because the reflectivity of different roads could cause large errors in laser measurements, it is difficult to use lasers for real vehicle applications of the LL detection system. High-precision maps can improve the accuracy of LL detection to a certain extent, but there are still some difficulties in mapping, which restrict the research and development of LL detection algorithms based on these sensors.

However, the sensor fusion algorithms, which depend on LiDAR, high-precision maps, and GPS, are complex, and the cost is higher than that of vision-based algorithms. Thus, the popularization and application of ADAS is blocked to a certain extent. Therefore, this study proposes an algorithm based on machine vision to study the LL high-precision detection in complex scenes in a low-cost way. At present, the lane detection method using visual information mainly uses three categories: Color-based, model-based, and learning-based methods.

The feature-based method does not need to consider the shape information of the road. Generally, according to the color, edge, or gradient change of LL and other feature information, the lane marking and the non-LL area on the road surface are separated by clustering or segmentation. Li et al. [21] delineated the region of interest in the captured image, eliminating noise using image graying and median filtering, while a spike neural network was used to detect the edge of the LL and the line itself was obtained using the Hough transform. Madrid et al. [22] proposed a lane departure warning system suitable for mobile devices, which used the Hough transform and image blurring to reduce the amount of calculations substantially and meet the real-time requirements. Gaikwad et al. [23] grayed the image and used a piecewise linear stretching function to improve the contrast of the interested region, achieving LL detection using the Hough transform. Yenİaydin et al. [24] proposed a robust lane detection algorithm using machine vision. However, grayscale images are greatly affected by some factors, such as shadows and the light intensity, and their use in lane detection is susceptible to environmental interference. After the image is converted to grayscale, color information is lost, resulting in the low robustness of the detection algorithm.

Detection methods based on color features can extract the color information in the road image effectively and obtain the LL information. Mammeri et al. [25] proposed an LL detection architecture, which combined the maximally stable extremal regions (MSER) algorithm and the Hough transform. By using MSER in a color image to determine the region of interest, and applying a three-level refined optimization algorithm to enhance the MSER results and filter out irrelevant information, such as trees and vehicles, the color and shape of LLs were matched. Nugraha et al. [26] converted the RGB color mode of the image into LUV and LAB color spaces, respectively, and extracted the yellow and white LLs on the road surface, achieving two-color LL detection after filtering. Comparing the aforementioned works with the LL detection algorithm using only grayscale images, we conclude that, to a certain extent, robustness is improved due to the use of color information. However, this grayscale information cannot be used to accurately perceive the changes in shape feature information and image size, so it is very difficult to detect local features.

In recent years, methods based on the convolutional neural network (CNN) [27,28,29] have also been gradually used for LL detection. Kim et al. [29] considered the global image information and transformed the detection of LLs into the task of region segmentation. Lee et al. [30] proposed an end-to-end trainable vanishing point guided network (VPGNet), which can obtain the position of the LL in the image by training a large number of pictures. Zhang et al. [16] proposed an LL detection algorithm based on the YOLO v3 using K-means clustering algorithm to optimize the network parameters. Kim et al. [31] proposed an LL detection scheme combining CNN and the random sample consensus (RANSAC) algorithm. Firstly, the image was input into the CNN network for denoising and enhancement after edge detection, and then the RANSAC algorithm was used to fit the LL. He et al. [32] input perspective and overhead images to CNN for training, respectively. The input of the network includes the image set of LL-positive samples (target LL) and the image set of LL-negative samples (road surface interference markers), and with the help of positive and negative labels, the network was trained. In order to solve the problem that the deep learning model needs a large number of label images, Zhang [28] et al. proposed a method using real scene images to synthesize label image sets for detecting lateral LL. The detection accuracy based on CNN is higher, because it uses a moving window to traverse the entire image, and the extracted features are the most abundant, but it also brings a huge amount of calculation, which greatly reduces the efficiency.

The length, width, and direction of LL have strong regularity, and the characteristics of serialization and structure association are obvious. The recurrent neural network (RNN) [33] is a kind of neural network that captures the dynamic information in serialized data through the periodic connection of hidden layer nodes. Ye et al. [34] proposed an LL detection method, which combines the CNN and long-term memory neural network. Li et al. [35] developed a multitask deep convolution network to detect the existence of the target and its geometric attributes (position and direction). Zou et al. [36] combined CNN and RNN, first extracted the feature information of each frame by CNN, and then input RNN for feature learning and LL prediction. Li [35] and Zou et al. [36] verified that RNN can successfully detect LLs in the case of missing LLs. Although RNN can improve the detection accuracy of LL by using continuous multi-frame information, the number of frames input into the network and the sampling interval between frames will affect the detection results, and multi-frame data is bound to increase the processing time. In this study, by using the spatial distribution law of LLs, a method of LL detection using LSTM and RNN is proposed. Only part of the known LL coordinate information is needed to estimate the position of other LLs.

## 3. Lane Line Prediction Algorithm

### 3.1. Recurrent Neural Network

**Figure 1:**
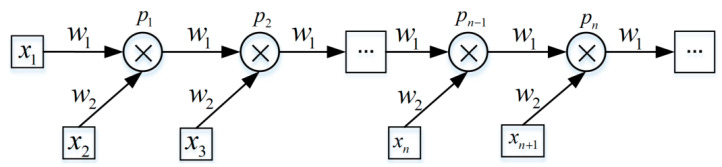
Structure of recurrent neural network (RcNN).

A recurrent neural network (RcNN) can construct a network structure recursively from the bottom to the top according to a given directed acyclic graph. In order to solve the problem that LLs are difficult to detect in the case of turns, we used the RcNN to represent lane line sequences with certain rules. By traversing the nodes in the directed acyclic graph topology, the vector of a child node is used to represent the vector of the learnt parent node. To unify and simplify the network structure, the directed acyclic graph was transformed to obtain a form of binary tree, as shown in Figure 1.

All nodes in the recurrent neural network have input data. For the ith node, the system state equation is:

(1) $$h^{\left(i\right)}=f\left(U^{T}h_{c}^{\left(i\right)}+W^{T}X+b\right),$$

where $h^{\left(i\right)},\;h_{c}^{\left(i\right)}$ represent the system state of the current node and its parent node, respectively; *X* denotes the input data of this node; *f* represents the activation function of the encapsulated feedforward neural network; and *U*, *W*, and *b* represent the weight coefficients. The recursive neural network can learn the distribution characteristics of LLs with a larger span by combining the characteristics of adjacent LLs. In the learning process, an LL sequence is formed according to the distribution relation of LLs, which not only retains LL feature information but also combines the hierarchical structure characteristics of LLs.

### 3.2. Lane Line Prediction Model Considering Angle Information

**Figure 2:**
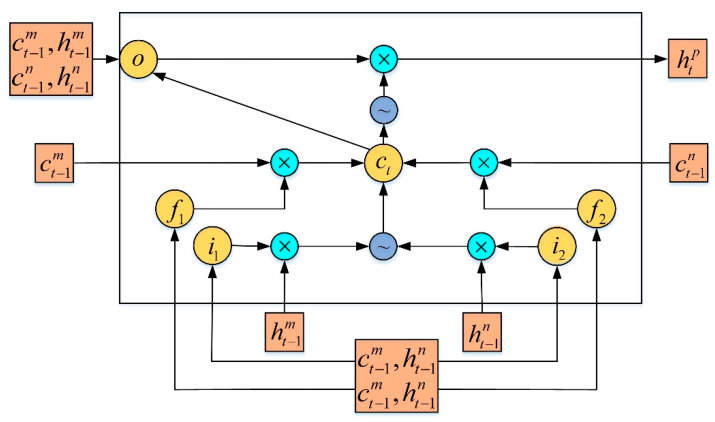
Network structure of Angle LSTM-RcNN (ALSTM-RcNN).

The recurrent neural network has obtained a good result for LL prediction on straight roads in previous works, but for curved lanes, it is difficult to accurately predict its position using only frame coordinate information. To solve this problem, the angle information must be taken into account. When constructing a deep neural network to train the lane detection model, the coordinate information of the LL should be calculated, and we should also consider whether there is an angle shift in the direction of the LL. This is because this shift is closely related to the direction of the front and rear LLs. In view of this structural phenomenon, an angle LSTM-RcNN (ALSTM-RcNN) based on an angular transfer model was constructed, as shown in Figure 2. The LSTM-RcNN unit has a binary tree structure composed of two input gates, one output gate, one memory unit, and two forgetting units. When calculating the vector of a parent node, the position and direction information of the two child nodes are taken into account in the ALSTM-RcNN model, which is proposed in this paper.

Similar to LSTM, the memory unit of ALSTM-RcNN is also composed of an input gate, forget gate, output gate, and memory cell. However, since ALSTM-RcNN is based on a tree structure, there is more than one input of LSTM-RcNN at time t (for binary tree, there are two inputs), so ALSTM-RcNN has two input gates and forget for each LSTM unit gate, as shown in Figure 2, and an ALSTM-RcNN cell based on the binary tree structure is composed of two input gates, one output gate, one memory cell, and two forget cells. The sign ‘~’ in Figure 2 represents the activation function ‘tanh’.

**Figure 3:**
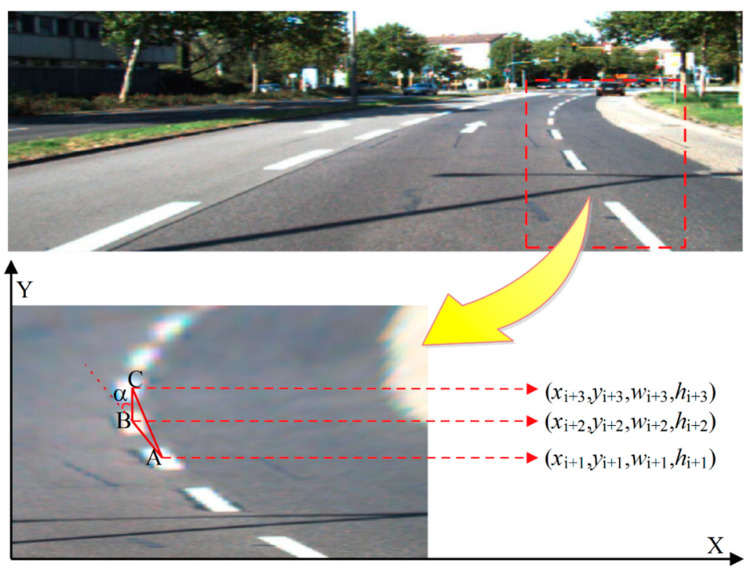
The angle of lane lines (LLs).

An LL steering angle example is shown in Figure 3, which shows a discontinuous LL composed of multiple LL blocks. The original image is mapped into a bird’s eye image and excludes irrelevant information. *A*, *B*, and *C* are the center coordinate points of the bounding boxes of the three LL blocks in the map. The cosine theorem can be used to determine the angle transfer α of *CB* to *AB*.

For driving safety, the angle of the LL is usually limited to a range to avoid sudden large-angle steering corrections. Considering that the method should try to include as many cases as possible, the range of α was limited to [−15°, 15°], with an interval of 2°, so that an angle transfer label set was obtained as A = {−15, −13, …, 0, …, 13, 15}. We defined the direction label of the left child of the current node as $\alpha_{l}$, that of the right child as $\alpha_{r}$, and the angle transfer vector as $\alpha_{*}^{lr}$. The vector of the current node is represented by the position and direction information of its two children. When training this model, a sequence containing the LL coordinates and the steering angle is input to the ALSTM-RcNN network, and the distribution rule of LLs is learned through the training model. Let the child nodes be *m* and *n*, respectively, while the parent nodes are denoted using *p*. $h_{t-1}^{m}$, $h_{t-1}^{n}$ represent the vectors of the child nodes in the hidden layer; $c_{t-1}^{m},\;c_{t-1}^{n}$ represent the memory vectors of the child nodes; ***P*** and ***Q*** are coefficient matrices; and $b_{*}$ is the bias term. The LL prediction model considering the angle information can be expressed as:

(2) $$i_{1}=\delta \left(P_{i1}^{m}h_{t-1}^{m}+P_{i1}^{n}h_{t-1}^{n}+Q_{i1}^{m}c_{t-1}^{m}+Q_{i1}^{n}c_{t-1}^{n}+b_{i1}\right),$$

(3) $$i_{2}=\delta \left(P_{i2}^{m}h_{t-1}^{m}+P_{i2}^{n}h_{t-1}^{n}+Q_{i2}^{m}c_{t-1}^{m}+Q_{i2}^{n}c_{t-1}^{n}+b_{i2}\right),$$

(4) $$f_{1}=\delta \left(P_{f1}^{m}h_{t-1}^{m}+P_{f1}^{n}h_{t-1}^{n}+Q_{f1}^{m}c_{t-1}^{m}+Q_{f1}^{n}c_{t-1}^{n}+b_{f1}\right),$$

(5) $$f_{2}=\delta \left(P_{f2}^{m}h_{t-1}^{m}+P_{f2}^{n}h_{t-1}^{n}+Q_{f2}^{m}c_{t-1}^{m}+Q_{f2}^{n}c_{t-1}^{n}+b_{f2}\right),$$

(6) $$c_{t}=f_{1}⊙c_{t-1}^{m}+f_{2}⊙c_{t-1}^{n}+\tanh\left(P_{c}^{x}h_{t-1}^{x}⊙i_{1}+P_{c}^{y}h_{t-1}^{y}⊙i_{2}+b_{c}\right),$$

(7) $$o=\delta \left(P_{o}^{m}{}_{t-1}^{m}+P_{o}^{n}{}_{t-1}^{n}+Q^{m}c_{t-1}^{m}+Q^{n}c_{t-1}^{n}+Q^{n}c_{t}+b_{o}\right),$$

where $i_{1}$, $i_{2}$ indicate the input gate; $f_{1}$, $f_{2}$ denote the forgetting gate; *o* indicates the parent node output gate; $c_{t}$ indicates the parent node’s forgetting gate; and $h_{t}^{p}$ indicates the output of the parent node.

## 4. Integration of Lane Detection Results Based on D-S

**Figure 4:**
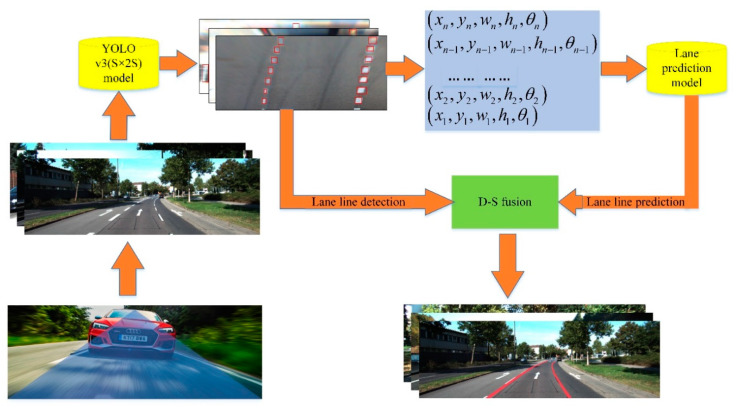
LL detection framework.

D-S evidence theory [37] is a kind of imprecise reasoning theory proposed by Dempster Shafer, which satisfies the weaker condition than Bayesian probability theory. It has unique advantages in uncertain information representation and multi-source evidence fusion. The basic principle is the uncertain information describing the system is transformed into evidence, and then the D-S evidence combination rule is used to fuse the multi-source uncertain evidence to form the mass function value of the identification framework Θ. Therefore, in this study, for the sake of improving the detection accuracy of the LL and avoiding the low precision caused by the false and weak detection of certain types of detectors (YOLO v3(S × 2S) and ALSTM-RcNN), the D-S evidence theory is used to fuse the output results of the two detectors and optimize the detection result. For the sake of improving the detection accuracy of the LL and avoiding the low precision caused by the false and weak detection of certain types of detectors, the D-S evidence theory was used to fuse the output results of the two detectors and optimize the detection result. The LL detection framework using the D-S evidence theory [30] is shown in Figure 4. The image collected by the camera is detected by the YOLO v3(S × 2S) model, and the lane position of LL is obtained. By constructing the LL sequence, the training of the prediction model is completed, and then the D-S evidence theory is used to integrate the output results of the YOLO v3(S × 2S) and the prediction model to obtain the optimal LL position information in complex scenes.

In D-S evidence theory, if *Ω* is the problem to be discerned, all possible and mutually incompatible decision results form a non-empty set Θ, which is the recognition framework of *Ω* [34]. In the present study, we determined whether there is an LL in a certain area, so the recognition framework can be expressed as $\Theta =\left\{\omega_{lane},\omega_{nolane}\right\}$. We defined the set function as *m*:$2^{\Theta }\to \left[0,1\right]$, which represents the basic assignment probability in Θ, satisfying the following equation:

(8) $$\{\begin{array}{l} m\left(\emptyset \right)=0\\ \sum_{A\subseteq \Theta }m\left(A\right)=1 \end{array},$$

where *m*(*A*) represents the trust degree of the focus element *A* and ∅ represents the empty set. In terms of two independent pieces of evidence, it was assumed that *m_1_* and *m_2_* are the distribution functions of the corresponding basic probabilities in Θ, and the focal elements are $A_{1},\ldots ,A_{M}$ and $B_{1},\ldots ,B_{M}$, respectively. According to the D-S evidence theory, we can synthesize the equation of the basic probability distribution function as follows:

(9) $$\{\begin{array}{l} m\left(\emptyset \right)=0\\ m\left(C\right)=\sum_{A_{i}\cap B_{j}=C}\frac{m_{1}\left(A_{i}\right)m_{2}\left(B_{j}\right)}{1-K},C\neq \emptyset \end{array},$$

where $K=\sum_{A\cap B=\emptyset }m_{1}\left(A_{i}\right)m_{2}\left(B_{j}\right)$ represents the uncertainty factor and reflects the degree of conflict between different evidence. The basic probability assignment (BPA) given by *m* is called the orthogonal sum of *m*_1_ and *m*_2_, which is $m_{1}⊕m_{2}$. If *K* = 1, then $m_{1}⊕m_{2}$ does not exist and the basic amplitude function cannot be synthesized.

In order to better manage the evidence conflict problem in DS theory, reliability is introduced by assigning a weight coefficient to the output of the two detectors. Reliability can reflect the quality of the fused information quantitatively, as information with high credibility has a higher weight and conversely for low credibility. This approach can enhance the advantages of the credible information selected, and improve the stability and reliability of the system during analysis. The performance of YOLO v3 (S × 2S) and ALSTM-RcNN are tested on the test set and the average accuracy rate is used as the credibility, recorded as *R*1 and *R*2, respectively. *A*_1_, …, *A*_k_ and *B*_1_, …, *B*_k_ are observed and assigned $1-R_{*}$ to the recognition frame *U* as unknown information. According to D-S theory, the combination rule is as follows:

(10) $$m\left(C\right)=\{\begin{array}{l} \frac{\sum_{\begin{array}{c} i,j\\ C \end{array}}\left[R_{1}m_{1}\left(A_{i}\right),\left(1-R_{1}\right)\right]\left[R_{2}m_{2}\left(B_{j}\right),\left(1-R_{2}\right)\right]}{1-K},\;\forall C\subset U,C\neq \emptyset \\ 0\;\;\;\;\;\;\;\;\;\;\;\;\;\;\;\;\;\;\;\;\;\;\;\;\;\;\;\;\;\;\;\;\;\;\;\;\;\;\;\;\;\;\;\;\;\;\;\;\;\;\;\;\;,\;C=\emptyset \end{array},$$

where $K=\sum_{\begin{array}{c} i,j\\ A_{i}\cap B_{j}=\emptyset \end{array}}\left[R_{1}m_{1}\left(A_{i}\right)\right]\left[R_{2}m_{2}\left(B_{j}\right)\right]$, $C=\left[A_{i},\left(1-R_{1}\right)\right]\cap \left[B_{i},\left(1-R_{2}\right)\right]$.

The decision-making based on the minimum risk is to find the minimum one in the decision set as the optimal decision-making. According to the calculation process of LL detection and the detection results of YOLO and LSTM-RcNN, the decision-making based on the basic probability assignment was adopted in this paper. The rules were as follows:

Suppose $\exists A_{1},A_{2}\subset U$,

If

(11) $$\{\begin{array}{l} m\left(A_{1}\right)=max\left\{m\left(A_{i}\right),A_{i}\subset \Theta \right\}\\ m\left(A_{2}\right)=max\left\{m\left(A_{i}\right),A_{i}\subset \Theta \;\&\;A_{i}\neq A_{1}\right\} \end{array},$$

and:

(12) $$\{\begin{array}{l} m\left(A_{1}\right)-m\left(A_{2}\right)>\varepsilon_{1}\\ m\left(\varphi \right)<\varepsilon_{2}\\ m\left(A_{1}\right)>m\left(\Theta \right) \end{array},$$

Then, *A*_1_ is the judgment result, where $\varepsilon_{1}$ and $\varepsilon_{2}$ represent preset thresholds, while *A*_1_ represents the result of the judgment.

## 5. Result Analysis and Discussion

The computer operating system used for testing was Ubuntu 14.04, the CPU was i7-7600K, with 16Gb of RAM and an NVIDIA GTX1080. We used Euro Truck Simulator 2’s photo gallery for training and testing, as it contains a wealth of traffic scenes, including a collection of night, rain, and fog images.

### 5.1. Model Training and Result Analysis

#### 5.1.1. Model Training Based on Transfer Learning

**Figure 5:**
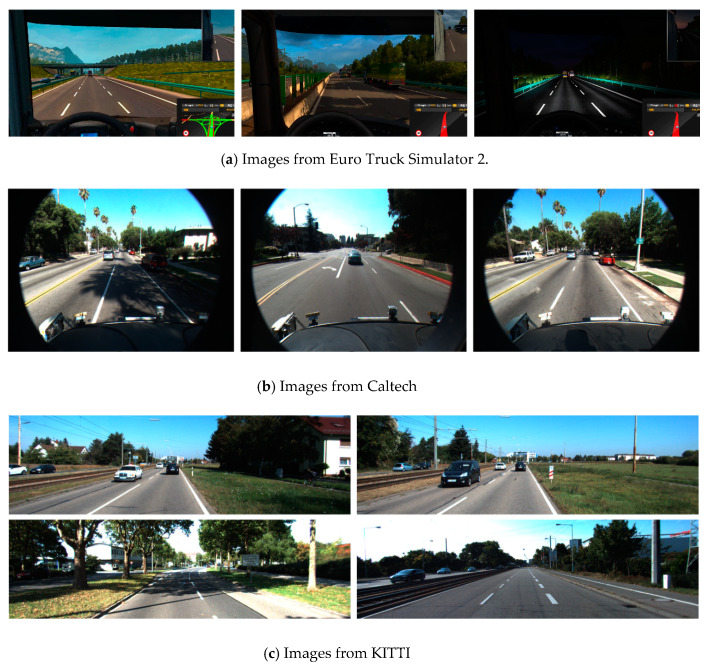
Three traffic scene images.

The transfer learning method [38,39] was used to train on the Euro Truck Simulator 2 (ETS2) traffic scene image set using the YOLO v3 (S × 2S) LL detection model. As shown in Figure 5, photo galleries including different weather conditions, such as night, rainy, and foggy images, were used. Caltech and KITTI traffic scene pictures were the source domains [40]. The YOLO v3 (S × 2S) LL detection model was trained using these two data sets. The task of transfer learning was to identify LLs in ETS2 traffic scenes, and the traffic scenes were the target areas.

**Figure 6:**
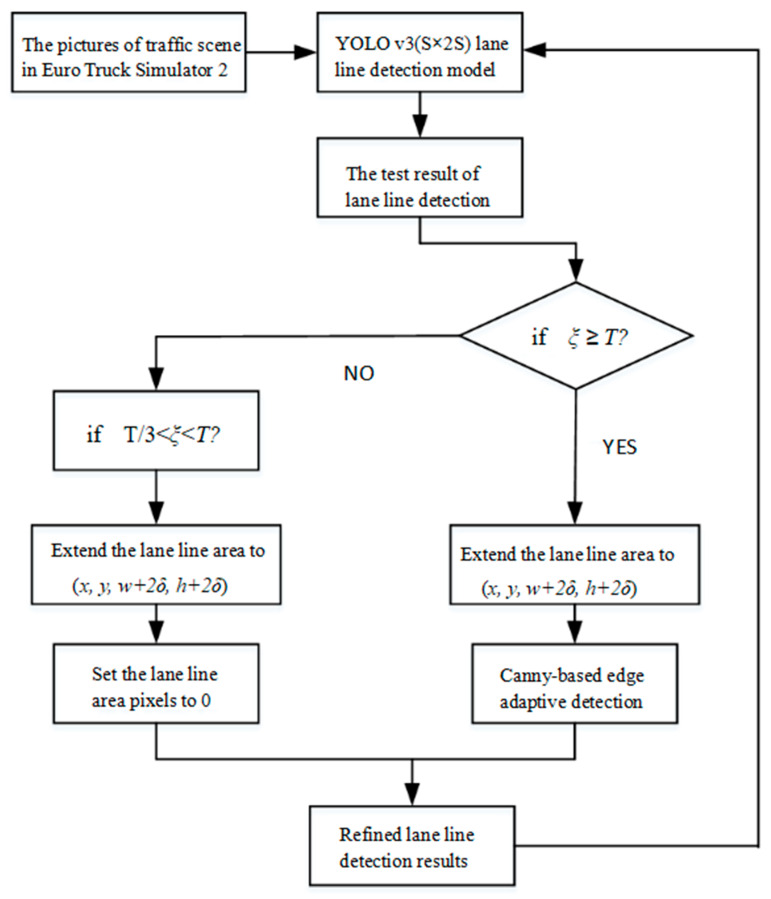
Training process of lane detection model based on the improved You Only Look Once (the third version) (YOLO V3(S × 2S)).

ETS2 has many traffic scenarios, which basically cover all weather types. In this study, sunny, rainy, and snowy weather was selected. The road types were mainly urban roads, highways, tunnels, and bridges. In order to improve the training efficiency, the screen recording software Camtasia Studio 8 was used to record the game process as a video file at 36 frames per second. The online training method was still used during training. The pictures collected from ETS2 traffic scenes were input to the YOLO v3 (S × 2S) model. The total time of the training video was 16 h and 26 min. The training parameters were initialized. The initial parameter values for training were set as follows: *learning_rate* = 0.001, *batchsize* = 128, *momentum* = 0.9. During the training process, the model’s detection capability was gradually enhanced, so we set a growth factor ζ = 2e−5 for T0. The training process is shown in Figure 6.

The picture of the game scene was input to the YOLO v3 (S × 2S) model. Initially, the detection was not ideal, so the detection results needed to be pre-processed so that they could be used as standard label pictures for model training. Meanwhile, using only the condition *T* ≤ *ξ* to confirm whether an LL is recognized will cause some LLs to be missed. During model training in the Caltech and KITTI scenes, because the predicted values with low confidence were probably non-LLs, the pixel value of the bounding box area of the confidence value of the detection result in the range of *T/4* < *ξ* < *T* was set to 0. Due to the lower interference in the ETS2 traffic scene images, and to accelerate the training speed, the threshold of the confidence value was increased from the original *T/4* to *T/3*, namely, the LL boundary box in the range of *T/3* < *ξ* < *T* was expanded to (*x*, *y*, *w* + *2δ, h + 2δ*), and the pixels in this area were set to 0. For bounding boxes whose confidence value satisfied *T* ≤ *ξ*, they were expanded to (*x, y, w + 2δ, h + 2δ*), and then an adaptive edge detection algorithm based on Canny [41] was used to quickly relocate the detected LLs.

**Figure 7:**
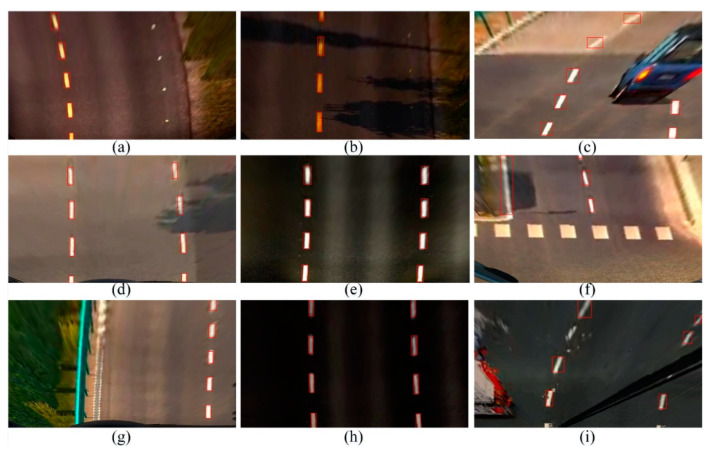
The results detected obtained using YOLO v3(S × 2S) in Euro Truck Simulator 2 (ETS2). The (**a**,**d**,**g**) are the detection result in scenes with good lighting conditions. The (**b**,**e**,**h**) are in the night. The (**c**,**f**,**i**) are in complex scenes such as vehicle occlusion, the interference of the zebra crossing, and rainy weather.

The detection results of the YOLO V3 (S × 2S) LL recognition model in ETS2 traffic scene images are shown in Figure 7. In Figure 7a,d,g, the model achieves better recognition results in scenes with good lighting conditions. Figure 7e,h show the results of the test conditions at night, where images of the road surface containing clear LLs are accurately identified under the illumination of the headlights. In Figure 7c, the YOLO v3 (S× 2S) LL detection model cannot predict the position of the LL using machine vision due to vehicle occlusion. In Figure 7f, despite the interference of the zebra crossing, the obtained model can distinguish the LL accurately, so the trained model is proved to have strong robustness. Figure 7i is a game scene in rainy weather. As the raindrops hit the windshield, the spray was generated, which caused the LL in the picture to be deformed. In the process of moving the wiper, part of the LL was also blocked, so some LLs could not be detected accurately.

To evaluate the performance of the YOLO v3 (S × 2S) LL recognition model, manual labeling was used to label the pictures in the Euro Truck Simulator 2 traffic scenes, and a standard data set used for testing was obtained and classified according to weather conditions, as shown in Table 1.

As shown in Table 1, the accuracy on the ETS2 traffic scene images reached 91% on sunny and cloudy scenes, exceeding the accuracy rates obtained on KITTI and Caltech due to the game scenes, in which the LLs were clearly marked and there was almost no interference from other obstacles. The accuracy of the sunny and cloudy scenes is the same, which indicates that the model’s detection of the LL is almost unaffected by the light intensity. In the rainy scenes, due to the interference of raindrops on the front windshield and the swing of the wipers, the shape of the LL in the image is deformed, which reduces the accuracy of LL recognition.

#### 5.1.2. Model Training for ALSTM-RcNNs

**Figure 8:**
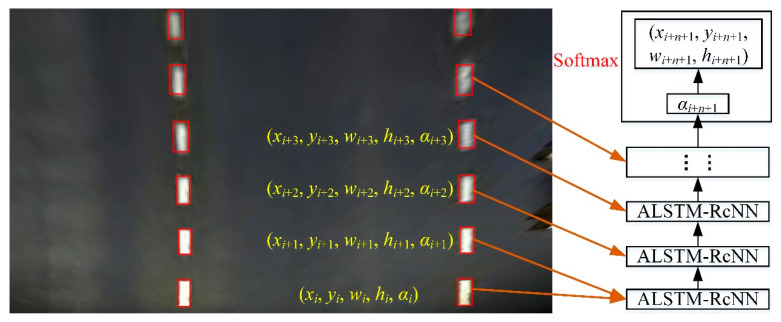
Training flow of the ALSTM-RcNN.

The analysis in the previous section showed that the LL detection model of LSTM-RcNN does not consider the angle information of the LL under steering conditions, which leads to a low LL prediction accuracy. In the process of constructing the ALSTM-RcNN model, the angle of each LL module is fully considered through the training of the network model, and the angle and coordinate information of the LLs are output using the softmax function. The YOLO v3 (S × 2S) LL detection model was used to detect LLs on the road surface and remove poorly performing pictures. We relocated the detected LLs using the Canny-based adaptive edge detection algorithm, and the steering angle of the LL was obtained using the cosine algorithm, so the parameters of each LL were (*x_i_*, *y_i_*, *w_i_*, *h_i_*, *α_i_*), where (*x_i_*, *y_i_*) are the center point coordinates of the bounding box, and *w_i_* and *h_i_* represent the width and height of the bounding box. To determine the images sets accurately identified, the LL area was expanded to (*x_i_*, *y_i_*, *w_i_* +*2δ, h_i_* +*2δ*). Using the adaptive Canny edge detection algorithm, the detected LL was relocated to obtain accurate coordinate information. After the preprocessing was completed, the optimized LL detection results were input into the ALSTM for model training. The training process is shown in Figure 8. The parameters of each LL were input into the ALSTM-RcNN model, and the Adam optimization method was used for training. The initial values of the training parameters were set as follows: *learning_rate* = 0.006, *batchsize* = 128. Through the feedback training network, the corresponding angle was output through the softmax layer, so that the coordinate information of the LL was obtained and the prediction of the LL in the case of the curve was realized.

**Figure 9:**
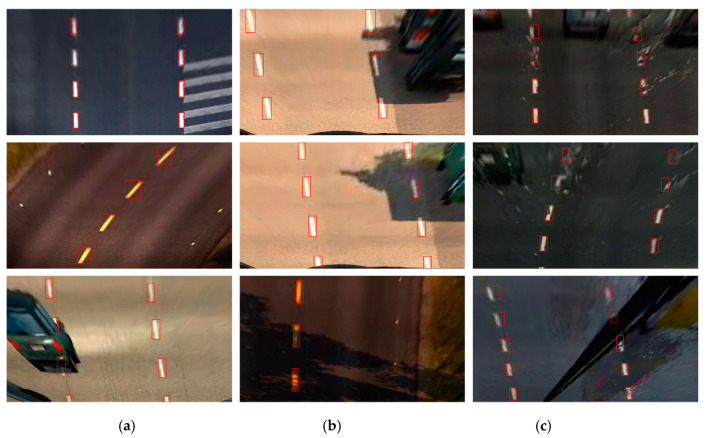
ALSTM-RcNN detection results on ETS2. The (**a**–**c**) are the lane detection result in the nighttime, shadow, and rain scenes, respectively.

The LL detection results using the ALSTM-RcNN model are shown in Figure 9. In nighttime (Figure 9a) and shadow scenes (Figure 9b), LLs were detected accurately. In Figure 9c, the LLs obtained were severely deformed due to the water droplets hitting the front windshield; however, the ALSTM-RcNN model still predicted them accurately.

The accuracy rate in each scenario is shown in Table 2, and is higher than the LSTM-RCNN model, with the MAP reaching 70%. The algorithm can realize LL detection under steering conditions. Although accurate detection cannot be performed when there is a change in the steering angle, the predicted LL coordinate information is more similar to the standard data set, thus the accuracy of traffic line identification is improved.

### 5.2. Results Analysis of Lane Line Detection

The ALSTM-RcNN uses the known LL position information and considers the angular variation of the LL in the curve situation, where the LL prediction is performed in the case of limited vision. The MAP of the LL detection model based on YOLO v3 (S × 2S) reaches 90%, which has a high detection accuracy. However, it is difficult to detect the LL when the LL is deformed due to occlusions or raindrops.

In order to comprehensively consider the identification results of two kinds of LL detectors and make the output LL coordinate information reach the optimum, D-S evidence theory was adopted to integrate the detection results of YOLO v3(S × 2S) and ALSTM-RcNN to obtain the final output accurate LL position. The identification framework of D-S evidence theory is $\Theta =\left\{\omega_{lane},\omega_{nolane}\right\}$, which is divided into two cases, depending on whether an LL exists or not. If an LL exists, the boundary box of the LL will be output. The YOLO detector detects the position of the LL using visual information, and the output value is (*ξ*_YOLO_, *x*, *y*, *h*, *w*). ALSTM-RcNN predicts the position center coordinates of the LL using existing LL position information, and ultimately outputs the maximum coordinates (*x*, *y*) of the confidence value *ξ*_RcNN_ using the softmax function. Considering the confidence value as the LL detection accuracy, the probability of LL presence is *ξ*_YOLO_ and *ξ*_RcNN_, respectively, and the probability of LL absence is 1−*ξ*_YOLO v3(S × 2S)_ and 1−*ξ*_ALSTM-RcNN_, respectively. To obtain the reliability of the two detectors, we used the images of the ETS2 image collection as test data to determine the reliability of YOLO v3 (S × 2S) and ALSTM-RcNN, as shown in Table 3. The accuracy of the ALSTM-RcNN algorithm by itself is significantly lower than that of the YOLO v3 (S × 2S) algorithm. However, since it can predict the LL position information in the case of rainy days and obstacles, after D-S evidence theory fusion, the MAP value of LL recognition is increased to 93.74%, reaching a high recognition level.

**Figure 10:**
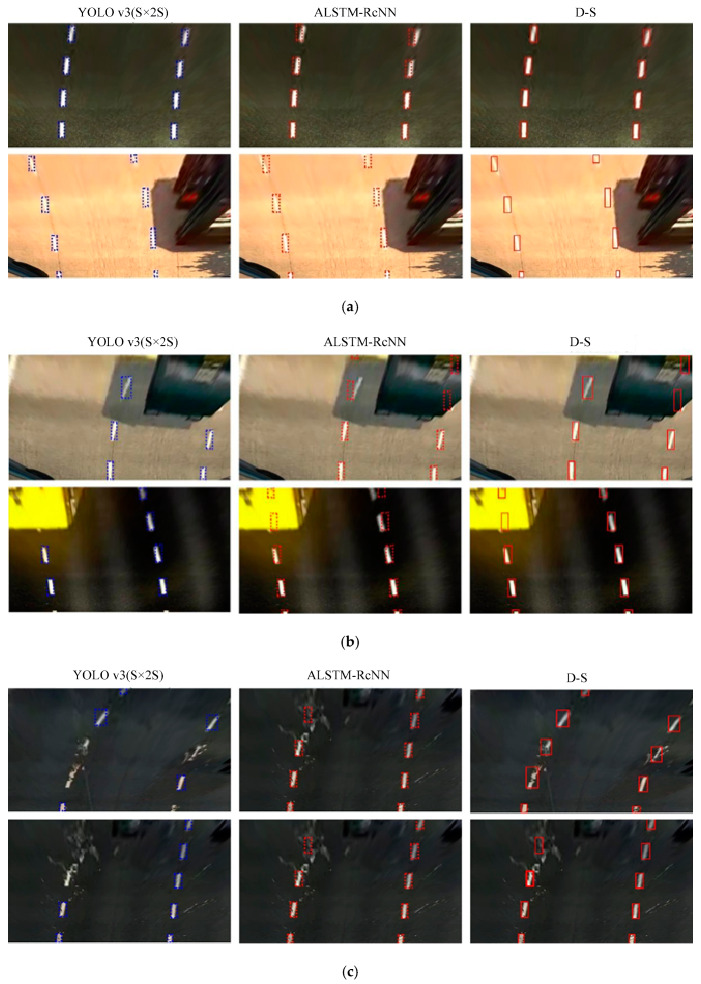
Detection results of YOLO v3 (S × 2S), ALSTM-RcNN, and Dempster Shafer (D-S) evidence theory. The (**a**) are the lane detection result in the scenes without obstacle occlusion; (**b**) are the result in the scenes with obstacle occlusion; (**c**) are in the rain scene.

The LL detection results using YOLO v3 (S × 2S), ALSTM-RcNN, and D-S fusion are shown in Figure 10. The YOLO v3 (S × 2S) model can detect the position of the LL without obstacle occlusion accurately, while the ALSTM-RcNN algorithm predicts the future LL sequence using the detected LL, making the recognition accuracy of the line slightly higher. After fusing the D-S evidence theory, the accurate LL position is output, as shown in Figure 10a. Part of the LL in Figure 10b is blocked by other vehicles, and the LL in Figure 10c is affected by raindrops. The LL obtained using the camera was severely deformed. The YOLO v3 (S × 2S) model can only detect visible LLs and not occluded and severely deformed LLs. The ALSTM-RcNN algorithm uses information on existing LLs to predict LL presence. Finally, the D-S evidence theory fuses the results of the two detectors and outputs accurate LL position information.

**Figure 11:**
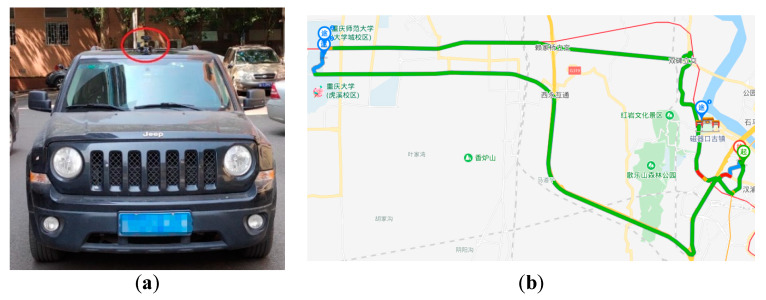
The layout of the camera and the driving route. The (**a**) is the layout of camera; (**b**) is the driving route.

To test the accuracy of the lane recognition algorithm, a set of system devices was designed using the real vehicle. The camera was placed at the upper center position of the windshield using a suction cup to collect the traffic images in front of the vehicle. The roads in the experiment were highways, urban streets, and tunnels marked with obvious LLs, and also included vehicle congestion and unobstructed scenes. Because the designed algorithm used machine vision, the brightness had a great influence on the detection results, so the test scene in this study contained the road conditions under different brightness. The experiment was carried out in Chongqing. The layout of the vehicle sensors and driving route is shown in Figure 11. Moreover, Caltech was introduced to analyze the detection results of LLs and verify the effectiveness of the algorithm by comparison.

In order to illustrate the effectiveness of the algorithm, the average accuracy (mAP) of the detected LL bounding box and the detection speed were used as evaluation criteria. The algorithm in this paper was compared with the detection results of current LL detection algorithms, such as Fast-RCNN [42], Faster-RCNN [43], Sliding window + CNN [44], SSD [45], and Context + RCNN [46]). The results are shown in the Table 4. Since the algorithm in this study adopted the method of combining YOLO v3(S × 2S) and ALSTM-RcNN, it has strong robustness to the detection of LLs in complex scenes, but because of its use of the RNN network, the detection speed is slower.

In addition, due to the complexity of road conditions in the real scene, the accuracy of LL detection is lower than that in Caltech. However, the algorithm in this study adopts the method of fusion of YOLO v3(S × 2S) and ALSTM-RcNN, which can predict the location of the lane line in complex scenes, such as LL wear and occlusion. Therefore, compared with the single YOLO v3(S × 2S) method, the accuracy of LL detection is greatly improved. However, in the Caltech data set, the LLs in the scenes of Cordoval and Washington are relatively clear, and only YOLO v3(S × 2S) can achieve better detection results. The algorithm in this study does not improve the accuracy much. In summary, the LL algorithm proposed in this paper combining YOLO v3(S × 2S) and ALSTM-RcNN has strong robustness in harsh scenarios.

**Figure 12:**
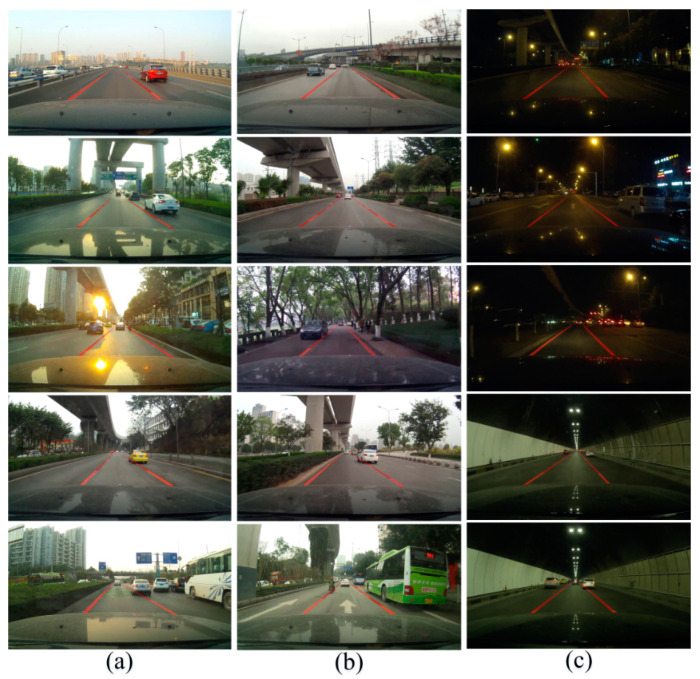
The LL detection result in real scenes. The LLs were detected in three scenarios, where (**a**) is on a sunny day, (**b**) is a cloudy day, and (**c**) is the detection result in tunnel and night scenes.

The LL bounding boxes detected by the algorithm in this study are independent of each other, but in the actual LL detection, a continuous line is needed. Therefore, the K-means and RANSAC algorithm were combined, and each individual LL bounding box was fitted into a curve. The final LL detection results are shown in Figure 12. In cloudy and sunny scenes, when the road conditions are good and there are no shadows or obstructions, the LL can be accurately detected. While in the night and tunnel scenes, the light source in the picture is mainly the lights and streetlights, which are darker than in the daytime, and the image quality collected by the camera at night is poor. The method in this study can predict the blurred and obscured LLs through the detected LLs, and output the complete LL results, which has strong robustness.

## 6. Conclusions

To solve the problem that the LLs are difficult to detect when occluded or deformed, the distribution of LLs and an LL detection method based on sequence information were studied. The LL prediction model ALSTM-RcNN with angle polarity was proposed. The tests showed that ALSTM-RcNN can predict the position of the LL accurately in both straight and curved situations. To improve the accuracy of LL detection and decrease the large errors in the recognition algorithm of single LLs, we used D-S evidence theory to integrate the output of the LL detection model YOLO v3 (S × 2S) and LL prediction model ALSTM-RcNN. The results showed that the detection accuracy of the LL after D-S fusion is significantly higher than the single detector.

However, though the experiments showed that the research method in this paper has high detection accuracy, due to the complexity of the algorithm, it cannot meet the real-time requirements. In future research, the multi-sensor fusion method will be considered to enable LL detection’s removal of visual restrictions. In complex scenes, not only accuracy but also real-time LL detection should be guaranteed.

## Figures and Tables

**Table 1:** Distribution and mean average precision (MAP) of label images.

Weather	Number	MAP
sunny	400	91.7%
cloudy	400	91.5%
rainy	300	86.4%

**Table 2:** MAP of ALSTM-RcNN.

Weather	MAP
Sunny	69.7%
Cloudy	70.4%
Rainy	68.4%

**Table 3:** The confidence value of ALSTM-RcNN on ETS2.

Algorithm	Confidence Value
YOLO v3 (S × 2S)	90.7%
ALSTM-RcNN	69.4%
D-S evidence theory	93.74%

**Table 4:** The detection accuracy and speed of algorithms on real scene images.

Algorithm	mAP	Time/ms
Fast RCNN	45.71%	2271
Faster RCNN	53.37%	122
Sliding window + CNN	64.82%	79,000
SSD	70.26%	29.3
Context + RCNN	73.43%	197
Yolo v3(S × 2S)	83.94%	25.2
The proposed algorithm	85.45%	124

## References

[1] Sun X., Cai Y., Wang S., Xu X., Chen L., Long C. (2019). Optimal control of intelligent vehicle longitudinal dynamics via hybrid model predictive control. Robot. Auton. Syst..

[2] Tang X., Zhang D., Liu T., Khajepour A., Yu H., Wang H. (2019). Research on the energy control of a dual-motor hybrid vehicle during engine start-stop process. Energy.

[3] Brinkley J., Posadas B., Sherman I., Daily S., Gilbert J.E. (2019). An open road evaluation of a self-driving Vehicle human–machine interface designed for visually impaired users. Int. J. Hum. Comput. Int..

[4] Li L., Wang X., Shao Y., Li W., Song F. (2018). Entropic pressure between fluctuating membranes in multilayer systems. Sci. China Phys. Mech. Astron..

[5] Zhang X., Yang W., Zhang Y., Liu J., Zhou S. An Improved Algorithm for Image Synthesis based on Gradient and Database. Proceedings of the 3rd International Conference on Robotics, Control and Automation (CACRE).

[6] Butakov V., Ioannou P. (2014). Personalized driver/vehicle lane change models for ADAS. IEEE Trans. Veh. Technol..

[7] Baskar L., Hellendoorn J., De Schutter B., Papp Z. (2011). Traffic control and intelligent vehicle highway systems: A survey. IET Intell. Transp. Syst..

[8] Lee H., Tomizuka M. (2003). Adaptive vehicle traction force control for intelligent vehicle highway systems (IVHSs). IEEE Trans. Ind. Electron..

[9] Tang X., Yang W., Hu X., Zhang D. (2017). A novel simplified model for torsional vibration analysis of a series-parallel hybrid electric vehicle. Mech. Syst. Signal Process..

[10] Zhang X., Yang W., Tang X., He Z. (2018). Estimation of the lateral distance between vehicle and lanes using convolutional neural network and vehicle dynamics. Appl. Sci..

[11] Wang R., Ye Q., Cai Y., Wang Y., Xu X., Meng X., Long C. (2018). Analyzing the influence of automatic steering system on the trajectory tracking accuracy of intelligent vehicle. Adv. Eng. Softw..

[12] Deng G., Wu Y. Double Lane Line Edge Detection Method based on Constraint Conditions Hough Transform. Proceedings of the 2018 17th International Symposium on Distributed Computing and Applications for Business Engineering and Science (DCABES).

[13] Tang X., Hu X., Yang W., Yu H. (2018). Novel torsional vibration modeling and assessment of a power-split hybrid electric vehicle equipped with a dual-mass flywheel. IEEE Trans. Veh. Technol..

[14] Gao L., Guo Z., Zhang H., Xu X., Shen H.T. (2017). Video captioning with attention-based LSTM and semantic consistency. IEEE Trans. Multimed..

[15] Redmon J., Farhadi A. Yolov3: An Incremental Improvement. https://arxiv.org/abs/1804.02767.

[16] Zhang X., Yang W., Tang X., Liu J. (2018). A fast learning method for accurate and robust lane detection using two-stage feature extraction with YOLO v3. Sensors.

[17] Shin S., Shim I., Kweon I.S. Combinatorial Approach for Lane Detection using Image and LIDAR Reflectance. Proceedings of the 2015 12th International Conference on Ubiquitous Robots and Ambient Intelligence (URAI).

[18] Rose C., Britt J., Allen J., Bevly D. (2014). An integrated vehicle navigation system utilizing lane-detection and lateral position estimation systems in difficult environments for GPS. IEEE Trans. Intell. Transp. Syst..

[19] De Paula Veronese L., Ismail A., Narayan V., Schulze M. An Accurate and Computational Efficient System for Detecting and Classifying Ego and Sides Lanes using LiDAR. Proceedings of the 2018 IEEE Intelligent Vehicles Symposium (IV).

[20] Jung J., Bae S.-H. (2018). Real-time road lane detection in urban areas using LiDAR data. Electronic.

[21] Li X., Wu Q., Kou Y., Hou L., Yang H. Lane Detection Based on Spiking Neural Network and Hough Transform. Proceedings of the 2015 8th International Congress on Image and Signal Processing (CISP).

[22] Madrid N., Hurtik P. (2016). Lane departure warning for mobile devices based on a fuzzy representation of images. Fuzzy Sets Syst..

[23] Gaikwad V., Lokhande S. (2014). Lane departure identification for advanced driver assistance. IEEE Trans. Intell. Transp. Syst..

[24] Yenİaydin Y., Schmidt K.W. A Lane Detection Algorithm Based on Reliable Lane Markings. Proceedings of the 2018 26th Signal Processing and Communications Applications Conference (SIU).

[25] Mammeri A., Boukerche A., Tang Z. (2016). A real-time lane marking localization, tracking and communication system. Comput. Commun..

[26] Nugraha B.T., Su S.F. Towards Self-Driving Car using Convolutional Neural network and Road Lane Detector. Proceedings of the 2017 2nd International Conference on Automation, Cognitive Science, Optics, Micro Electro-Mechanical System, and Information Technology (ICACOMIT).

[27] Cao Y., Chen Y., Khosla D. (2014). Spiking deep convolutional neural networks for energy-efficient object Recognition. Int. J. Comput. Vis..

[28] Zhang X., Yang W., Tang X., Wang Y. (2019). Lateral distance detection model based on convolutional neural network. IET Intell. Transp. Syst..

[29] Kim J., Kim J., Jang G.-J., Lee M. (2017). Fast learning method for convolutional neural networks using extreme learning machine and its application to lane detection. Neural Netw..

[30] Lee S., Kim J., Yoon J.S., Shin S., Bailo O., Kim N., Lee T.-H., Hong H.S., Han S.-H., Kweon I.S. VPGNet: Vanishing Point Guided Network for Lane and Road Marking Detection and Recognition. Proceedings of the IEEE International Conference on Computer Vision (ICCV).

[31] Kim J., Lee M. Robust Lane Detection Based on Convolutional Neural Network and Random Sample Consensus. Proceedings of the International Conference on Neural Information Processing (ICNIP 2017), Montreal Convention Center.

[32] He B., Ai R., Yan Y., Lang X. Accurate and Robust Lane Detection based on Dual-View Convolutional Neutral Network. Proceedings of the 2016 IEEE Intelligent Vehicles Symposium (IV).

[33] Mishra A.K., Desai V. (2006). Drought forecasting using feed-forward recursive neural network. Ecol. Model..

[34] Ye Y.Y., Hao X.L., Chen H.J. (2018). Lane detection method based on lane structural analysis and CNNs. IET Intell. Transp. Syst..

[35] Li J., Mei X., Prokhorov D., Tao D. (2017). Deep neural network for structural prediction and lane detection in traffic scene. IEEE Trans. Neural Netw. Learn. Syst..

[36] Zou Q., Jiang H., Dai Q., Yue Y., Chen L., Wang Q. (2020). Robust lane detection from continuous driving scenes using deep neural networks. IEEE Trans. Veh. Technol..

[37] Ayoub N., Gao Z., Chen B., Jian M. (2018). A synthetic fusion rule for salient region detection under the framework of DS-evidence theory. Symmetry.

[38] Shin H.-C., Roth H.R., Gao M., Lu L., Xu Z., Nogues I., Yao J., Mollura D., Summers R.M., Hoo-Chang S. (2016). Deep convolutional neural networks for computer-aided detection: CNN architectures, dataset characteristics and transfer learning. IEEE Trans. Med. Imaging.

[39] Singh J., Hanson J., Paliwal K.K., Zhou Y. (2019). RNA secondary structure prediction using an ensemble of two-dimensional deep neural networks and transfer learning. Nat. Commun..

[40] Narote S.P., Bhujbal P.N., Narote A.S., Dhane D. (2018). A review of recent advances in lane detection and departure warning system. Pattern Recognit..

[41] Son J., Yoo H., Kim S., Sohn K. (2015). Real-time illumination invariant lane detection for lane departure warning system. Expert Syst. Appl..

[42] Zhang X., Zhuang Y., Wang W., Pedrycz W. (2017). Online feature transformation learning for cross-domain object category recognition. IEEE Trans. Neural Netw. Learn. Syst..

[43] Pan J., Sayrol E., Giro-I-Nieto X., McGuinness K., O’Connor N.E. Shallow and Deep Convolutional Networks for Saliency Prediction. Proceedings of the IEEE Conference on Computer Vision and Pattern Recognition (CVPR) 2016.

[44] Ahmed E., Clark A., Mohay G. (2008). A novel sliding window based change detection algorithm for asymmetric traffic. Proceedings of the 2008 IFIP International Conference on Network and Parallel Computing.

[45] Liu W., Anguelov D., Erhan D., Szegedy C., Reed S., Fu C.Y., Berg A.C. SSD: Single Shot Multibox Detector. Proceedings of the 14th European Conference on Computer Vision (ECCV) 2016.

[46] Tian Y., Gelernter J., Wang X., Chen W., Gao J., Zhang Y., Li X. (2018). Lane marking detection via deep convolutional neural network. Neurocomputing.

